# Modified Technique to Fabricate a Definitive Closed Bulb Hollow Obturator for Maxillectomy in a Patient Following COVID-Associated Mucormycosis: A Case Report

**DOI:** 10.7759/cureus.58468

**Published:** 2024-04-17

**Authors:** Alcina Fernandes, Grazina Fernandes, Kennedy Mascarenhas, Meena Aras, Vidya Chitre

**Affiliations:** 1 Department of Prosthodontics and Crown and Bridge, Goa Dental College and Hospital, Panaji, IND

**Keywords:** palatal obturator, rehabilitation, mucormycosis, maxillectomy, customized impression technique, closed bulb hollow obturator, hollow bulb obturator

## Abstract

Immunocompromised people developed mucormycosis as a result of the COVID-19 outbreak. Antifungal medications, surgical excision of infected tissues, and therapy of underlying metabolic problems are available forms of treatment. Usually, surgery entails completely excising the affected area. The patient is at risk for nasal twang, nasal cavity leaks, and impaired masticatory function because of these anomalies. The obturator prosthesis may form an oro-nasal seal in such problems. Additionally, lowering the prosthesis weight contributes to improved stability and retention. This case report explains a novel flasking technique to lessen the prosthesis weight and a modified impression technique to capture the palatal deformity.

## Introduction

There was a spike in the number of cases of mucormycosis, or "Black Fungus," that were either directly or indirectly linked to COVID-19 during the second wave of coronavirus disease, or COVID-19, infection caused by the severe acute respiratory syndrome coronavirus 2 (SARS-CoV-2) virus in the year 2021 worldwide. The standard prosthetic treatment of defects plays a significant role in developing function for the patient [1,2].

Rehabilitating a maxillary defect frequently requires the use of a maxillary obturator. Keeping the nasal and oral cavities apart to allow for appropriate deglutition and articulation, supporting the orbital contents to prevent enophthalmos and diplopia, supporting the soft tissue to restore the midfacial contour, and attaining a satisfactory esthetic result are the goals of rehabilitation for patients who have had a total or partial maxillectomy [3,4].

The present article describes the prosthetic rehabilitation of a postsurgical defect due to mucormycosis.

## Case presentation

**Figure 1:**
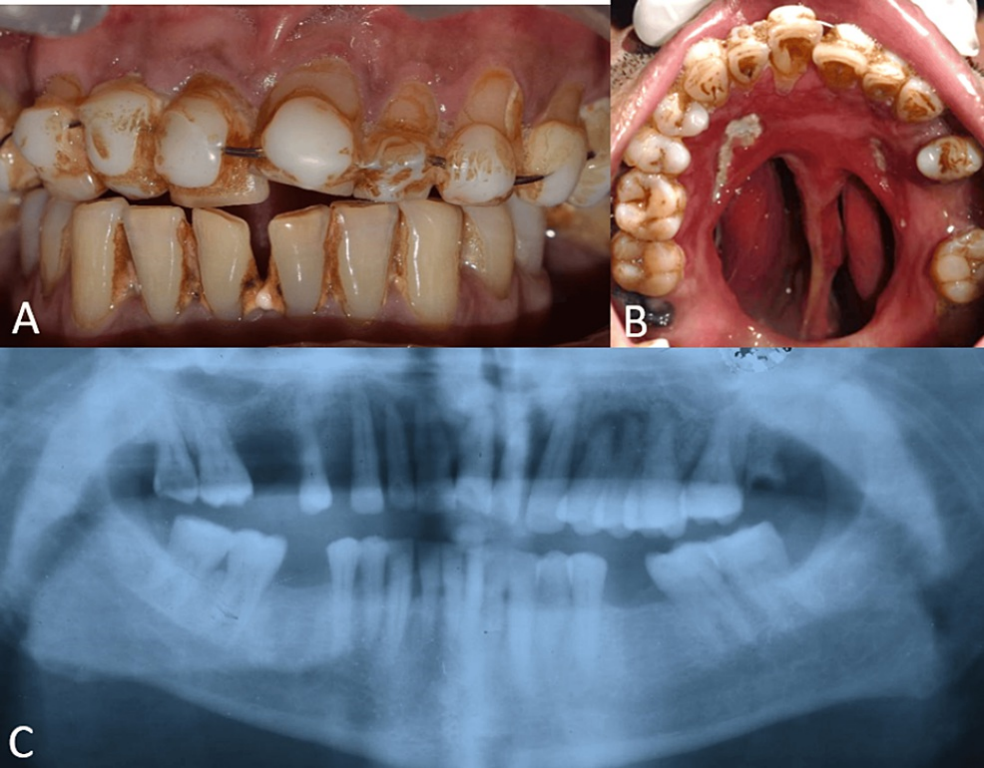
Intraoral and OPG view showing the maxillary defect (A) Frontal view on maximum intercuspation; (B) occlusal view of the maxillary arch showing the defect in the palate; (C) OPG showing the maxillary defect. OPG: Orthopantomogram

A 47-year-old male patient was referred to the Department of Prosthodontics for prosthetic rehabilitation. The patient gave a history of uncontrolled diabetes with COVID-19 infection which resulted in chronic invasive fungal rhinosinusitis with mucormycosis. He underwent diagnostic nasal endoscopy (DNE) and hard palate sequestral excision under local anesthesia. As a result, the hard palate was removed, resulting in an Aramany Class III maxillary deformity (Figure 1). Upon clinical examination, the remaining teeth and the anterior hard palate were found to be movable. A lightweight hollow closed bulb maxillary obturator was therefore suggested as part of the patient's treatment plan to help with speech, deglutition, and mastication.

Procedure

**Figure 2:**
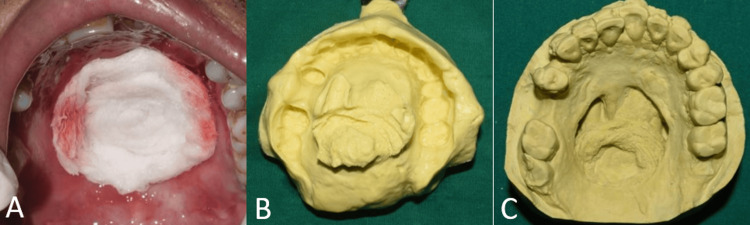
Primary impression and cast (A) Occlusal view of the maxillary arch showing the defect in the palate blocked with gauze; (B) primary impression of the maxillary arch; (C) primary cast of the maxillary arch

Primary impression was made with irreversible hydrocolloid (Vignette chromatic, Dentsply India Pvt Ltd, Bengaluru, India) by blocking the palatal defect with gauze (Figure 2).

**Figure 3:**
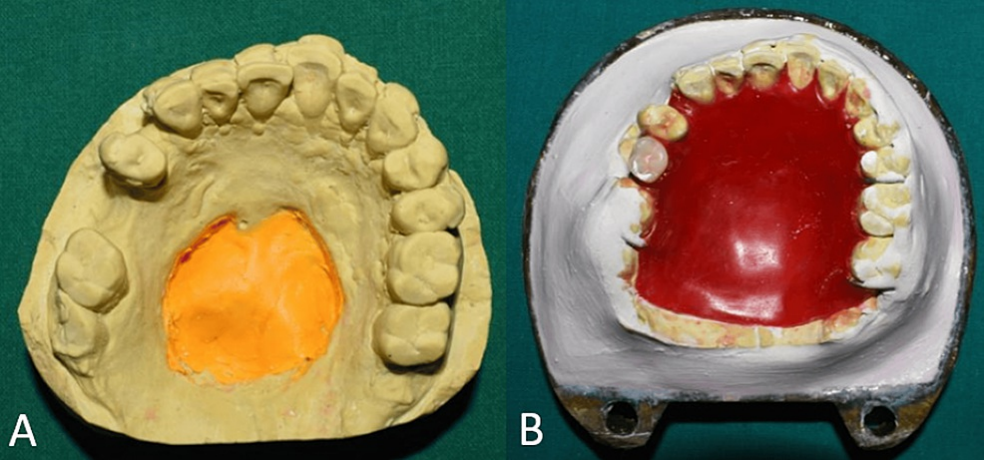
Lab procedures (A) Primary cast showing blocked undercuts; (B) investing for fabrication of interim obturator.

**Figure 4:**
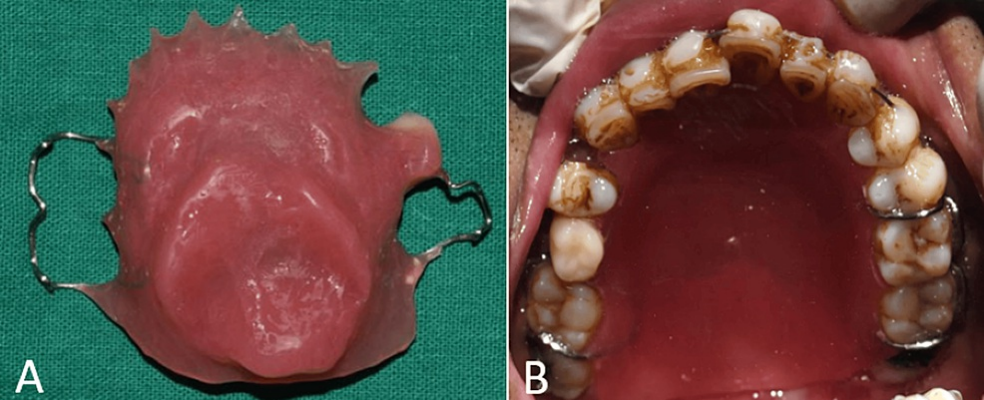
Interim obturator (A) Intaglio surface of the interim obturator; (B) occlusal view of the maxillary arch showing the interim obturator.

Impressions were poured and undercuts were blocked with putty silicone impression material (Zhermack Elite HD+, Zhermack SpA, Badia Polesine, Italy) (Figure 3). The interim obturator was fabricated incorporating a long-span Adams clasp on teeth no. 26 and 27 with an interproximal loop [5] for extra retention due to mobile teeth (Figure 4A). Conventional methods were used for dewaxing and investing. The interim obturator was delivered after finishing and polishing (Figure 4B).

For six months, the patient had weekly follow-ups. Once the patient's hard palate and teeth were less mobile, a definitive hollow bulb obturator was scheduled. The diagnostic cast was surveyed and the cast metal framework was designed with the following components: embrasure clasps on teeth no. 26 and 27; occlusal rest on teeth no. 15 and 17, cingulum rest on 13 and 23, modified complete palatal type of major connector extended till palatal surfaces of teeth. Distal surfaces of teeth no. 14, 17, and 27 were also prepared to act as a guiding plane.

**Figure 5:**
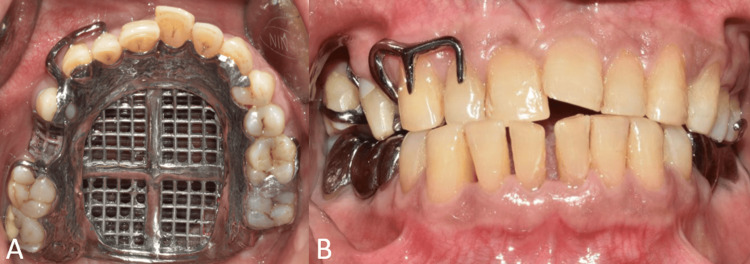
Intraoral view showing metal framework trial (A) Occlusal view showing metal framework trial; (B) frontal view showing metal framework trial.

Mouth preparation was done, and the mesio-occlusal rest seats were prepared on teeth no. 17, 24, and 27. The disto-occlusal rest seats were prepared on teeth no. 15 and 26. The canine rest seats were prepared on 13 and 23. Impression with polyvinyl siloxane (Elite HD+, Zhermack SpA, Badia Polesine, Italy) poured and cast metal framework was fabricated by conventional procedure. Cast metal framework in situ (Figure 5).

**Figure 6:**
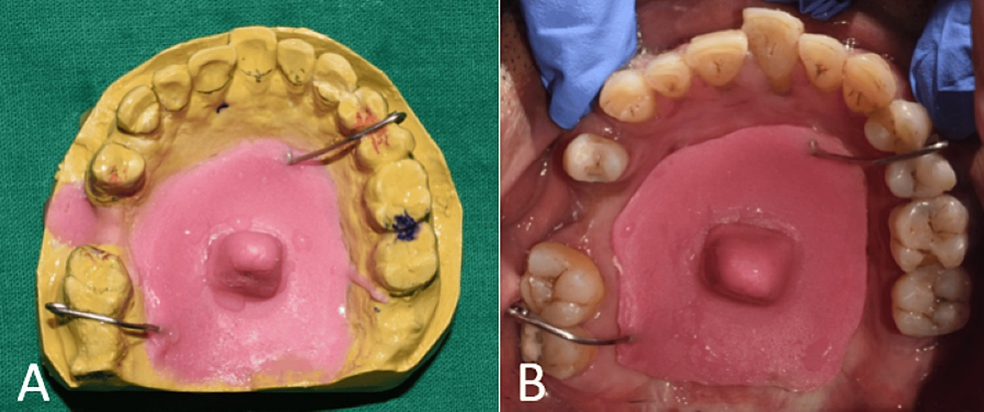
Modified impression technique (A) Primary cast showing custom acrylic tray for making the final impression; (B) intraoral view showing the custom acrylic tray.

A modified impression technique was used to capture the palatal defect, custom impression trays were fabricated using autopolymerizing acrylic resin (DPI-RR Cold CureTM, Dental Product of India, India) and adjusted to accommodate the palatal deformity by incorporating clasps for increased stability as shown in Figure 6.

**Figure 7:**
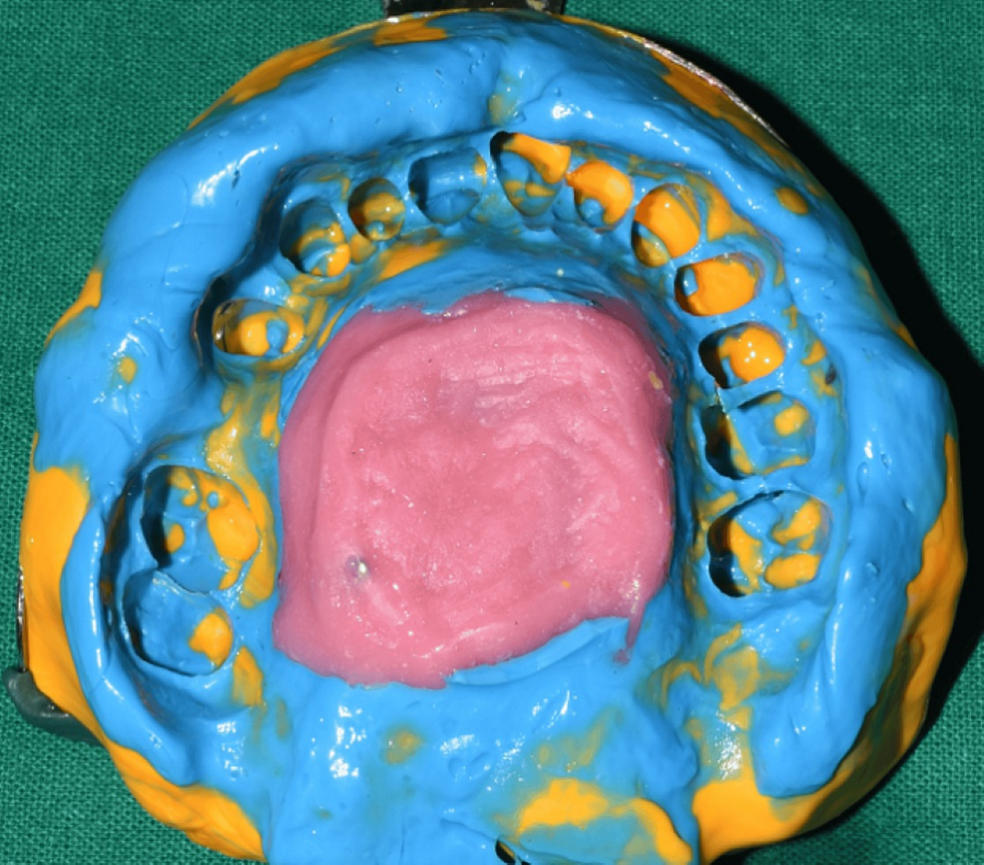
Final impression using the modified impression technique

**Figure 8:**
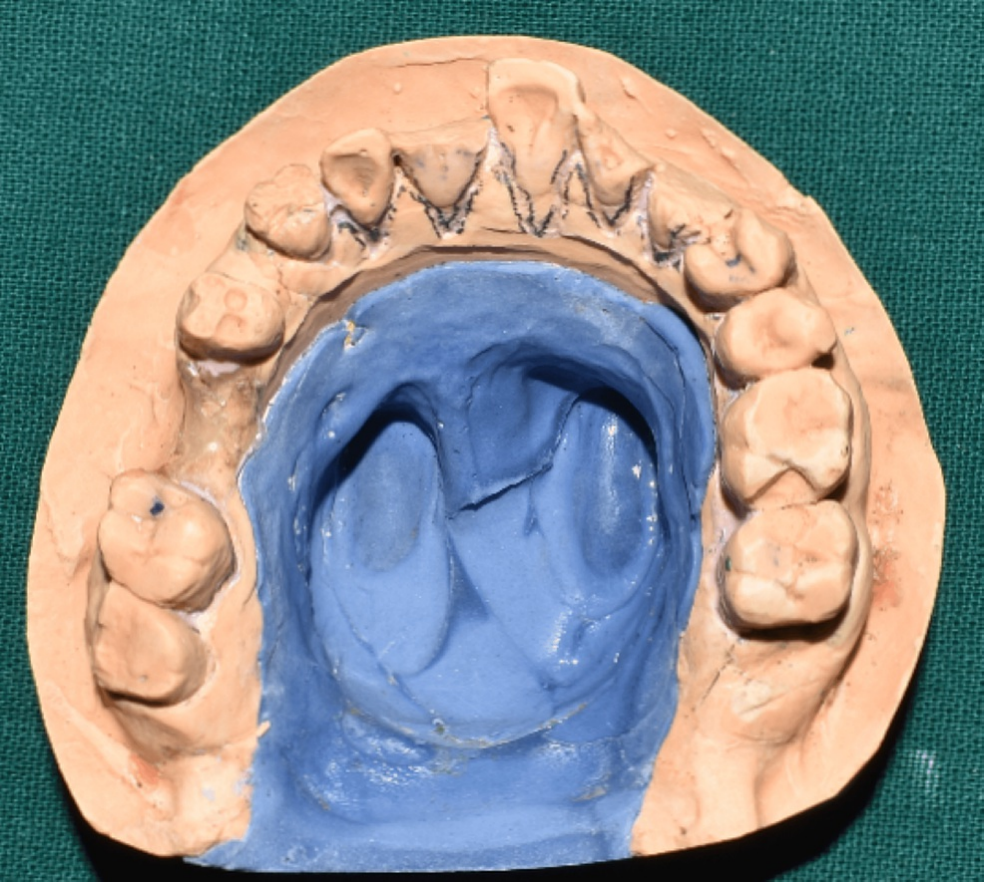
Altered cast

To make the impression, a stock tray covered in putty silicone impression material (Elite HD+, Zhermack SpA, Badia Polesine, Italy) was placed over a custom acrylic tray. Wash impression was made using light body polyvinylsiloxane (Elite HD+, Zhermack SpA, Badia Polesine, Italy). The final impression was adapted on the diagnostic cast and the cast was poured by using an altered cast technique (Figures 7, 8).

**Figure 9:**
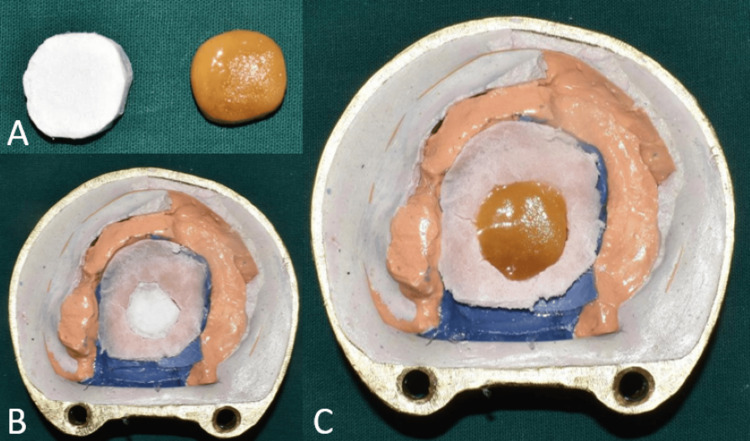
Fabrication of hollow obturator (A) Image showing thermocol and jaggery of similar sizes; (B) trial closure was done with thermocol placed in the defect; (C) thermocol was replaced by jaggery to fabricate the hollow obturator.

Investing and dewaxing was done. For fabrication of hollow closed bulb obturator thermocol (polystyrene sheet) was used as a spacer as it is inert and conforms to the shape of the defect without much deformation. Trial closure was done with thermocol filled in the defect area (Figure 9). Thermocol was replaced by jaggery (cane sugar) similar in shape and size as it is highly soluble, easy to retrieve, and moldable [6].

**Figure 10:**
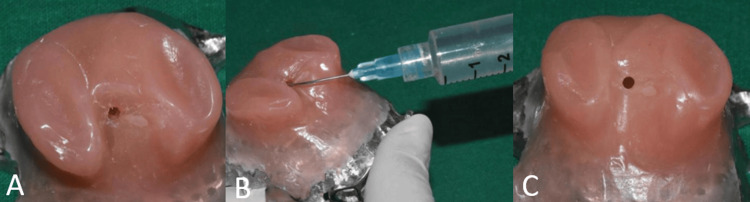
Procedure for removal of jaggery (A) A 2 mm hole made on the intaglio surface of the obturator; (B) a syringe used to flush out jaggery; (C) after removal of jaggery.

**Figure 11:**
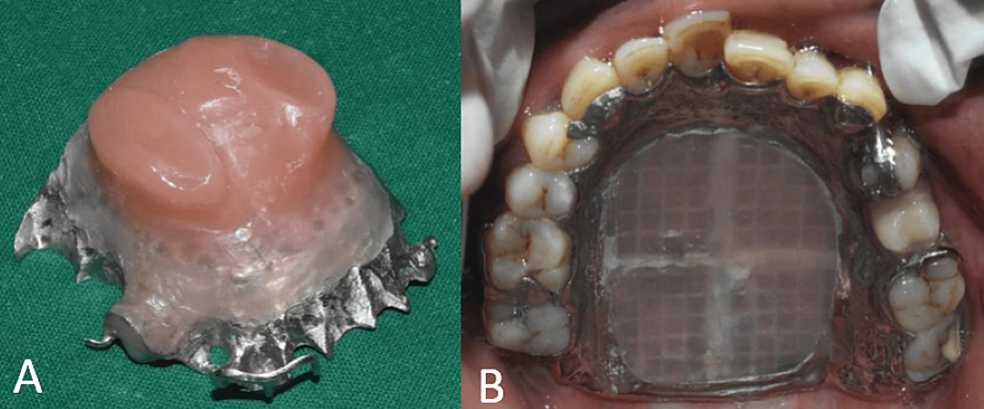
Final prosthesis (A) Intaglio surface of the obturator; (B) occlusal view showing the final prosthesis.

After acrylisation, a small hole of 2 mm diameter was made into the denture area for removal of jaggery. A syringe with warm water was used to flush out the jaggery from the defect area (Figure 10). Autopolymersing resin was used to pack the hole and the definitive prosthesis was finished and polished (Figure 11). The patient was given post-insertion instructions and educated regarding hygiene maintenance.

## Discussion

Mucormycosis is one of the rare opportunistic fungal infections presenting in different clinical forms. In 40% of individuals with mucormycosis, diabetes was shown to be the most prevalent predisposing condition. When the entire maxillary arch is affected, the lesion has an erosive character [7]. To prevent any recurrence, such patients typically have a very comprehensive bilateral maxillectomy. Patients' acceptance of one more intrusive treatment and inadequate bone are cited as reasons for the difficulty of surgical rehabilitation. Prosthetic rehabilitation using immediate, interim, and definitive obturators begins right after surgery if the defect is not surgically repaired. It preserves the midfacial contour by lifting and supporting soft tissues, promoting proper deglutition and speech articulation [8].

The classification of defects may lead to different framework designs for obturators. To limit dislodging functional forces, the prosthesis design should incorporate a robust primary connector for cross-arch stabilization as well as stabilizing and retaining components [9,10].

Many methods have been detailed for creating open and closed bulb hollow obturators, which reduce weight in the prosthesis such as putty, salt, and soap. To prevent fluid collection, the open hollow bulb obturator has to be cleaned frequently or have vent holes placed. It mostly accumulates fluids, mucus, and food [11]. Conversely, closed bulb obturators do not accumulate fluid since they extend sufficiently into the defect [12].

In this case report a novel technique for creating a closed hollow bulb obturator prosthesis has been detailed, which uses thermocol to regulate the hollow bulb's thickness and a jaggery spacer as methods used prior require multiple processing steps and are difficult to retrieve. Jaggery is a non-centrifugal cane sugar that is completely soluble in water. The benefits of using this technique include simplicity in manufacturing as the thermocol and jaggery can be shaped as needed, shorter time spent in the lab, the economy of scale, rigidity, and simplicity in retrieval [6].

## Conclusions

In terms of the patient's need for both esthetics and function, the single-piece fully hollow design used in this report proved to be an excellent rehabilitation technique and allowed us to maintain the prosthesis weight as low as feasible. A modified impression technique was used to record the palatal defect. The approach demonstrates how to create a patient-centered, cost-effective method using widely available materials.
